# Eye pigmentation–based in-ovo chicken sexing via precision breeding

**DOI:** 10.3389/fbioe.2026.1785893

**Published:** 2026-04-01

**Authors:** Yi-Chen Chen, Shuo-Wen Hsu, Eisuke Shimokita, Tatsuya Takemoto

**Affiliations:** 1 Division of Research and Development, Setsuro Tech Inc., Tokushima, Japan; 2 Department of Anatomy and Cell Biology, Tokushima University Graduate School of Medical Sciences, Tokushima, Japan; 3 Laboratory of Embryology, Institute of Advanced Medical Sciences, Tokushima University, Tokushima, Japan

**Keywords:** genome editing, in-ovo sexing, poultry, precision breeding, SLC45A2

## Abstract

The systematic culling of male layer chicks raises ethical concerns, leading to bans in Germany and other European countries and spurring the search for reliable in-ovo sexing methods. Most existing strategies rely on integration of exogenous DNA or are limited to specific chicken strains, and none meet commercial requirements. Here, we present a broadly applicable in-ovo sexing method that avoids exogenous DNA integration. We developed precision-bred chickens with targeted disruption of the Z-linked SLC45A2 gene, which encodes a transporter essential for pigmentation. Hemizygous knockout females (ZW; SLC45A2^KO/W^) exhibited eye depigmentation at embryonic day 7 (E7), whereas heterozygous knockout males (ZZ; SLC45A2^KO/+^) retained normal pigmentation. This clear visual dimorphism enables accurate sexing by routine egg candling. Fertility and reproductive performance of knockouts were comparable to wild-type chickens, and genotyping confirmed 100% prediction accuracy. Unlike many current technologies, our approach requires no complex instrumentation and allows early detection during incubation. This work provides a practical and ethical solution to chick sexing, with significant advantages for commercial hatcheries. More broadly, this study illustrates the potential of precision breeding to address pressing animal welfare concerns in the modern poultry industry.

## 1 Introduction

For decades, male chicks in the egg industry have been culled immediately after hatching once their sex has been confirmed, as they do not lay eggs and are inefficient for meat production. Annually, billions of male chicks are culled worldwide, and these practices have attracted widespread criticism from animal rights groups and the public, who argue that the mass killing of day-old chicks is inhumane (Krautwald-Junghanns et al., 2018). In July 2021, Germany and France, supported by Ireland, Spain, Luxembourg, Austria, and Portugal, requested an impact assessment from the European Commission for a potential EU-wide ban on the systematic killing of male chicks (Di Concetto et al., 2023). In 2020, both the German and French agricultural ministries committed to ending the practice by the end of 2021 and successfully implemented it. Reflecting global trends, France and Germany now require hatcheries to adopt in-ovo sexing technologies, which allow for sex determination within the egg.

Several methods for in-ovo sexing have been developed, including DNA- or compound-based analyses, immunosensing approaches, optical and non-optical spectroscopy, and genetic engineering strategies (Xie et al., 2023; Corion et al., 2023). However, the complexity of instrumentation, late testing windows, and the need for invasive sampling remain major obstacles to large-scale industrial implementation. These limitations highlight the need for a method that is both highly accurate and operationally simple for routine hatchery use.

Solute carrier family 45 member 2 (SLC45A2) encodes a transmembrane protein essential for melanosome maturation and pigmentation through regulation of melanosomal pH and tyrosinase activity (Bin et al., 2015). In chickens, SLC45A2 is located on the Z chromosome. Naturally occurring mutations in this gene have been associated with sex-linked imperfect albinism, characterized by pink eyes and diluted feather pigmentation (Gunnarsson et al., 2007), suggesting that this pigmentation phenotype is related to gene mutation, although direct functional validation has been limited. We therefore hypothesized that targeted disruption of SLC45A2 could produce a sex-dependent embryonic eye pigmentation difference, enabling early and non-invasive in-ovo sex identification. In this study, we tested this hypothesis by establishing a precision-bred SLC45A2 knockout chicken line and evaluating its suitability for practical in-ovo sexing applications.

## 2 Materials and methods

### 2.1 Experimental design and study overview

To evaluate the feasibility of eye pigmentation–based in-ovo chicken sexing, a stepwise workflow was implemented. Chicken primordial germ cells (PGCs) underwent targeted mutagenesis of SLC45A2 using a site-directed nuclease approach to generate knockout alleles. SLC45A2 knockout PGCs were introduced into recipient embryos to produce germline chimeras, which were subsequently bred to establish a SLC45A2 knockout chicken line. Next, embryonic eye pigmentation was then characterized during early development to identify a detectable sex-linked trait in the knockout chickens. Reproductive performance, including egg production, was evaluated to assess suitability for breeding applications. Finally, a pilot in-ovo sexing validation was performed to determine whether embryonic eye pigmentation could be reliably detected by simple candling as a non-invasive sexing method.

### 2.2 Chicken and chicken embryos

Fertile eggs were obtained from Okazaki Station, National Livestock Breeding Center, Japan. The fertile eggs of the Barred Plymouth Rock (BPR) strain of chicken (*Gallus gallus*) were used as PGC donor embryos, whereas the fertile eggs of the White Leghorn (WL) strain of chicken (*Gallus gallus*) were used as recipient embryos for PGC transfer. Fertile eggs and offspring generated from chimeras and SLC45A2 knockout chickens were produced via artificial insemination at Kyodoken Institute, Japan. Chicken embryos were incubated in a humidified egg incubator (Showafuranki, Japan) at 38 °C with automatic egg turning. Egg candling was performed with an LED egg candler (Showafuranki, Japan) in a dark room. Chickens were kept under a 15/9-h light/dark cycle, individually caged, and provided a commercial diet and water *ad libitum*. Egg-laying performance was monitored daily in three wild-type and three knockout hens to determine the onset of laying (age at first egg). Egg production between 28 and 33 weeks of age was recorded weekly and expressed as egg-laying rate (%), with a theoretical maximum of seven eggs per week (one egg per day) or 42 eggs over the 6-week period defined as 100%. Statistical analyses were performed using GraphPad Prism 9 (GraphPad Software, Inc., United States). Differences between groups were evaluated using an unpaired t-test, and statistical significance was defined as P < 0.05. All animal procedures were approved by the Institutional Animal Care and Use Committee of Tokushima University (T2021-53) and Kyodoken Institute (A2021-001-3).

### 2.3 Plasmid construction

A previously synthesized C-terminal nucleoplasmin NLS-conjugated MAD7 nuclease (nMAD7) expression plasmid containing an mScarlet reporter was utilized in this study (Chen et al., 2023). For crRNA cloning, a pair of BbsI restriction enzyme digestion sites were introduced immediately after the MAD7 crRNA sequence in the MAD7 expression plasmid, allowing annealed crRNA oligos (Supplementary Table S1) to be inserted via restriction cloning via BbsI and T4 ligases (New England Biolabs, United States). Candidate crRNAs were designed with CHOPCHOP (CRISPR/Cpf1) (Labun et al., 2019), and oligos were synthesized and procured from Eurofins Japan (Japan).

### 2.4 Cell culture and manipulation

Chicken PGCs were derived from established Barred Plymouth Rock (BPR) strain PGC stocks and maintained according to previously described methods and culture conditions (Chen et al., 2019; Chen et al., 2023). The derived PGC clones were stored at −80 °C in Bambanker freezing medium (NIPPON Genetics, Japan) for future use. For genetic modification, 1 µg of plasmid DNA was used for 5 × 10^4^ cells in a 10 µL electroporation reaction. Electroporation was performed under previously described conditions with a Neon Transfection System (Thermo Fisher Scientific, United States) (Chen et al., 2023). For PGC transplantation, 5 × 10^4^ PGCs were transplanted into the circulatory system of E3 recipient embryos in 5 µL of basic medium, following the method described by Chen et al. (Chen et al., 2019) for transplantation in the gonad migration assay. Experiments were conducted with more than three repetitions.

### 2.5 FACS and analysis

Fluorescent cell enrichment, single-cell sorting, and analysis were performed via a BD FACSMelody cell sorter (BD Biosciences, United States) with its default configuration. The instrument was equipped with blue (488 nm), red (640 nm), and yellow–green (561 nm) lasers and was operated with BD Chorus software (BD Biosciences, United States). All procedures were conducted following the manufacturer’s guidelines as outlined in the user manual.

### 2.6 DNA extraction, PCR, and amplicon sequence analysis

Genomic DNA (gDNA) samples were extracted from cells or tissues via the DNeasy Blood & Tissue Kit (Qiagen, United States). For amplicon sequence analysis, DNA fragments surrounding the crRNA target site were amplified through a two-step PCR process using specific primer sets (Supplementary Table S2) and Index PCR primers, as outlined in the manufacturer’s instructions (Illumina, United States). Following gel purification, the amplicons were sequenced via the MiSeq platform with the MiSeq Reagent Kit v2 (Illumina, United States). Sequence alignment of the amplicons was performed with SnapGene software (GSL Biotech, United States).

## 3 Results

### 3.1 Gene editing design for chicken SLC45A2 gene targeting

**FIGURE 1:**
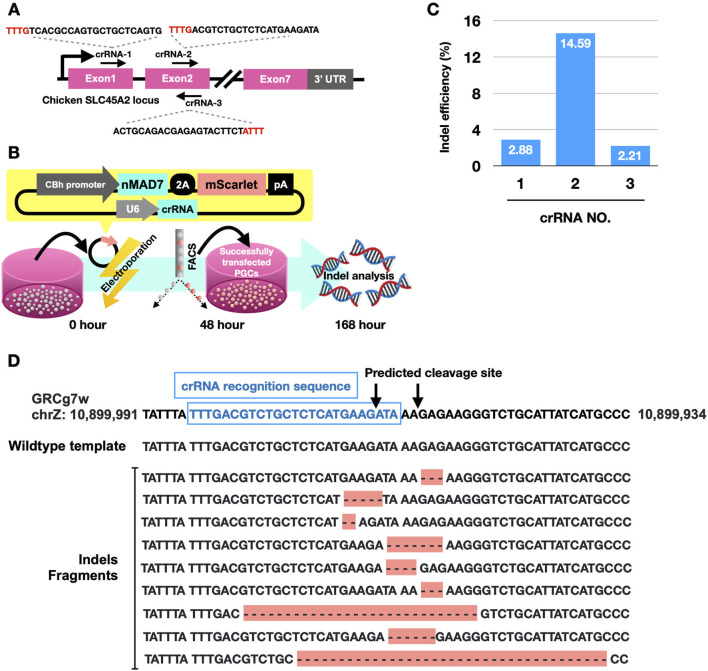
Chicken SLC45A2 gene-targeting design and evaluation via CRISPR-MAD7 in PGCs. **(A)** Three MAD7 crRNAs targeting the coding region of chicken SLC45A2 were designed, with each crRNA recognition sequence displayed and the PAM sites labeled in red. **(B)** Experimental procedure for plasmid transfection and indel formation analysis: Plasmids expressing nMAD7, crRNA, and mScarlet were electroporated into PGCs. The transfected cells were harvested via FACS for mScarlet-positive expression 48 h post-transfection. At 168 h post-transfection, DNA was extracted for indel analysis via amplicon sequencing via next-generation sequencing (NGS). **(C)** A bar chart showing the indel efficiency among the 3 crRNA candidates. **(D)** Sequencing data for PGCs electroporated with crRNA-2 revealed multinucleotide deletions (indels fragments) around the predicted cleavage site (arrow).

We first identified the crRNA showing the highest DNA cleavage activity for the SLC45A2 gene. We utilized MAD7 nuclease conjugated with a C-terminal nucleoplasmin NLS (nMAD7), which showed efficient gene-editing activity in chicken primordial germ cells (PGCs) in our previous study (Chen et al., 2023). As shown in Figure 1A and Table 1, we screened three crRNAs, one targeting exon 1 (crRNA-1) and two targeting exon 2 (crRNA-2 and crRNA-3) of the SLC45A2 locus. We introduced a plasmid expressing nMAD7 and each crRNA by electroporation into male PGCs. Successfully transfected PGCs were harvested via FACS 48 h post-electroporation, followed by prolonged culture until 168 h post-electroporation. Genomic DNA was then collected for amplicon sequencing analysis (Figure 1B). As shown in Figure 1C and Table 1, crRNA-2 presented the highest indel frequency (14.59%; 421/2885), with deletion patterns around the predicted cleavage site (Figure 1D). Thus, crRNA-2 was utilized for further gene editing to knock out the SLC45A2 gene.

**TABLE 1:** Designed crRNAs for chicken SLC45A2 and their indel formation abilities in PGCs.

crRNA NO.	Target sequence (5’ - 3′)	PAM	Genomic location (GRCg7w)	Strand	Efficiency^a^	Amplicon sequencing results		
						Indel read	Total read	Indel frequency (%)
1	TCA​CGC​CAG​TGC​TGC​TCA​GTG	TTTG	chrZ 10,901,410–10,901,434	-	29	91	3159	2.88
2	ACG​TCT​GCT​CTC​ATG​AAG​ATA	TTTG	chrZ 10,899,961–10,899,985	-	63	421	2885	14.59
3	TCT​TCA​TGA​GAG​CAG​ACG​TCA	TTTA	chrZ 10,899,959–10,899,983	+	56	36	1627	2.21

^a^ Efficiency was scored by CHOPCHOP, with the scoring method described by Kim et al. (2018).

### 3.2 SLC45A2 knockout PGCs and phenotypic evaluation in derivative chickens

**FIGURE 2:**
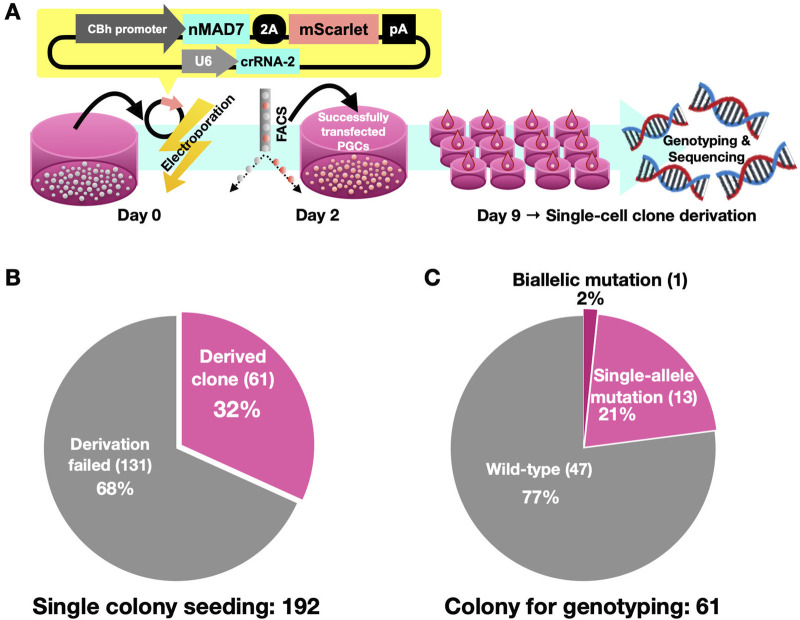
Derivation of SLC45A2 mutant PGC clones. **(A)** Illustration outlining the derivation process for SLC45A2-mutant PGC clones: nMAD7-expressing plasmids were electroporated into PGCs on Day 0. Transfected PGCs were enriched via FACS on Day 2, cultured until Day 9, and seeded as single cells into 192 wells. Successfully derived clones were analyzed for SLC45A2 sequences following propagation. **(B)** Of the 192 seedings, 61 (32%) developed into PGC clones, whereas 131 (68%) did not propagate. **(C)** Among the 61 clones, 47 had no mutation (wild-type, 77%), 13 had single-allele mutations (21%), and 1 had biallelic mutations in SLC45A2 (2%).

Next, we generated SLC45A2 knockout PGCs. We electroporated plasmids expressing nMAD7 and crRNA-2 into male PGCs. As shown in Figure 2A, successfully transfected PGCs expressing mScarlet were enriched via FACS on Day 2 post-electroporation. These pooled gene-edited PGCs were cultured until Day 9 and then seeded as single cells into 192 wells. Among the 192 single-cell colonies, 61 clones (32%) were obtained (Figure 2B). Genotyping revealed that 14 clones (23%) carried DNA deletions at the crRNA-2 target region, 13 of which presented single-allele deletions, and one clone (No. 24) presented biallelic deletions (Figure 2C; Table 2).

**TABLE 2:** DNA deletion and amino acid substitution in derived SLC45A2-mutant PGC clones.

Clone no.	Allele-1 deletion DNA codon	Allele-1 amino acid substitution	Allele-2 deletion DNA codon	Allele-2 amino acid substitution
5	-GAAGAT-	Deletion from 192E-193D (no frame shift)	Wild-type	-
15	-TAAAGA-	Deletion from 194K to 195E; D193E (no frame shift)	Wild-type	-
17	-GAAGATAAAG-	Deletion from 192F to 198K; E192R (frame shift)	Wild-type	-
21	-TGAAGATAA-	Deletion from 192E-193D; H191Q (no frame shift)	Wild-type	-
23	-TCT​CAT​GAA​GAT​AAA​GAG​AAG​GGT​CTG​C-	Deletion from 190S to 198L; S190I (frame shift)	Wild-type	-
24	-AAGCATATTTATTTGACGTCTGCTCTCATGAAGA TAAAGAGAAGGGTCTGCATTATCATGCCCTGTTT ACA-	Deletion from 183A to 205T; K182R (frame shift)	-GAAGAT-	Deletion from 192E-193D (no frame shift)
26	-GA-	E192R (frame shift)	Wild-type	-
28	-GA-	E195E (frame shift)	Wild-type	-
38	-GA-	E192R (frame shift)	Wild-type	-
43	-AAGA-	E192V (frame shift)	Wild-type	-
48	-GAAG-	E192I (frame shift)	Wild-type	-
55	-AGA-	E192D (no frame shift)	Wild-type	-
60	-TAAA-	D193E (frame shift)	Wild-type	-
62	-TAAAGA-	Deletion from 194K to 195E; D193E (no frame shift)	Wild-type	-

**FIGURE 3:**
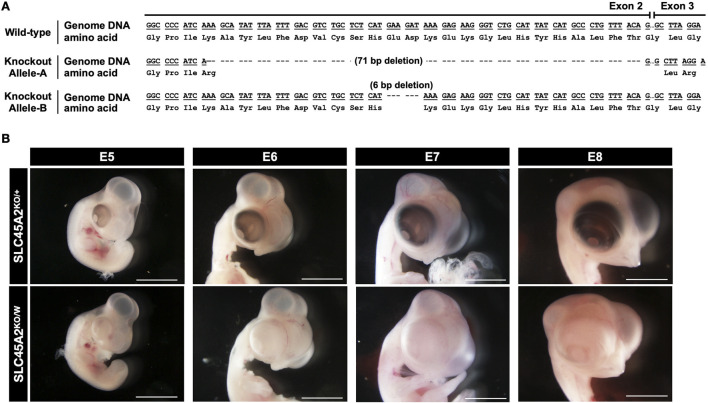
Genotype and phenotype of SLC45A2 knockout chickens. **(A)** The SLC45A2 knockout DNA sequences exhibited two distinct deletions in exon 2: a 71-bp deletion (Knockout Allele-A) and a 6-bp deletion (Knockout Allele-B). In Knockout Allele-A, the deletion induced a frame shift at exon 2. In contrast, no frame shift was predicted to occur in Knockout Allele-B. **(B)** Eye pigmentation phenotypes of SLC45A2 knockout heterozygous (♂; SLC45A2^KO/+^) and hemizygous (♀; SLC45A2^KO/W^) embryos from E5 to E8. Images were captured under BF illumination. Scale bar: 5 mm. Images are representative of more than three samples.

We selected the biallelic deletion mutant PGC clone No. 24, which carries 71-bp and 6-bp deletions, for further offspring production. Using the same transplantation method described in our previous study (Chen et al., 2018; Chen et al., 2023), we generated 11 F0 chimeras (7 males and 4 females). By intercrossing F0 chimeras and the wild-type, we successfully generated 4 heterozygous knockout F1 males (SLC45A2^KO/+^) in 30 F1 chicks from F0 chimeras. Genotypic sequencing confirmed that these heterozygous knockout F1 males carried a 71-bp deletion (Knockout Allele-A) in exon 2 of the SLC45A2 gene (Figure 3A). This deletion caused a frameshift in the amino acid sequence starting at position 182. On the other hand, no offspring carrying Allele-B knockouts were identified in the F1 generation, but we predicted that both mutant DNA sequences were heritable.

We used these heterozygous knockout F1 roosters to produce heterozygous knockout males (SLC45A2^KO/+^) and hemizygous knockout females (SLC45A2^KO/W^). Eye pigmentation phenotypes were evaluated in embryos from E5 to E8. Compared with heterozygous knockout males, hemizygous knockout female embryos did not exhibit eye pigmentation, indicating that the 71-bp deletion disrupted the function of SLC45A2 in pigmentation synthesis (Figure 3B). These results confirm that chicken SLC45A2 is essential for eye pigmentation and demonstrates sex linkage due to its location on the Z chromosome.

**FIGURE 4:**
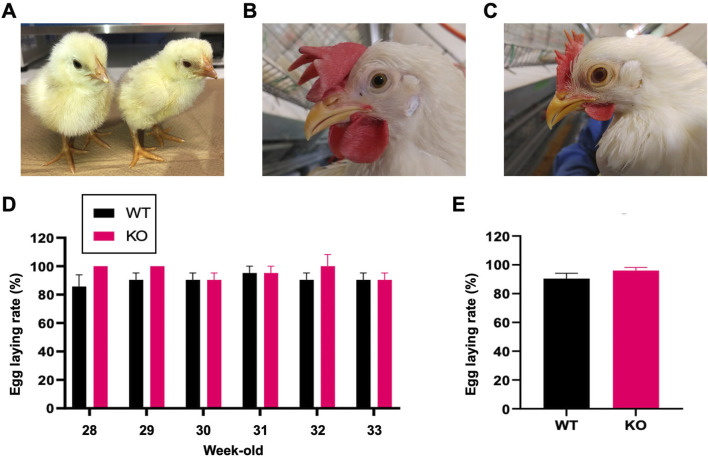
Phenotypic comparison and egg-laying performance of SLC45A2 wild-type and knockout chickens. **(A)** Wild-type (left) and knockout (right) chicks at the hatchling stage. **(B)** Head appearance of an adult SLC45A2 wild-type hen (28 weeks old). **(C)** Head appearance of an adult SLC45A2 knockout hen (28 weeks old). **(D)** Weekly egg-laying rates (%) of wild-type (WT) and knockout (KO) hens from 28 to 33 weeks of age, with a maximum of seven eggs per week (one egg per day) set as 100%. No statistically significant difference was observed between the two groups. The error bars represent the standard error of the mean (SEM) (t-test, P = 0.08, n = 3). **(E)** Average egg-laying rate over the 6-week period, with a maximum of 42 eggs (one egg per day) set as 100%. No statistically significant difference was observed. The error bars represent the SEMs (t-test, P = 0.2558, n = 3). Images are representative of more than three samples.

While we confirmed that SLC45A2 knockout chickens reproduce normally from the F1 to F3 generations (Supplementary Table S3), we assessed their egg-laying performance, a critical economic trait in poultry. Heterozygous knockout roosters were crossed with wild-type hens to produce hemizygous SLC45A2 knockout and wild-type females. At the hatchling stage, no discernible differences were observed between the wild-type and knockout chicks, except for lighter eye pigmentation in the knockout females (Figure 4A). In adult hens, eye color was indistinguishable between the two groups (Figures 4B,C). Egg laying began at 18 and 21 weeks in wild-type hens and at 18, 19, and 20 weeks in knockout hens (n = 3). The weekly egg-laying rates from 28 to 33 weeks were not significantly different (Figure 4D; Supplementary Table S4). The average egg-laying rate during this period was comparable between the knockout and wild-type hens (96.0% ± 2.1% vs. 90.5% ± 3.6%), indicating that SLC45A2 deletion does not affect reproductive performance (Figure 4E). These findings suggest that while SLC45A2 influences pigmentation, it does not impair reproductive ability.

### 3.3 Practical application of in-ovo sexing in the SLC45A2 knockout-derived eggs

**FIGURE 5:**
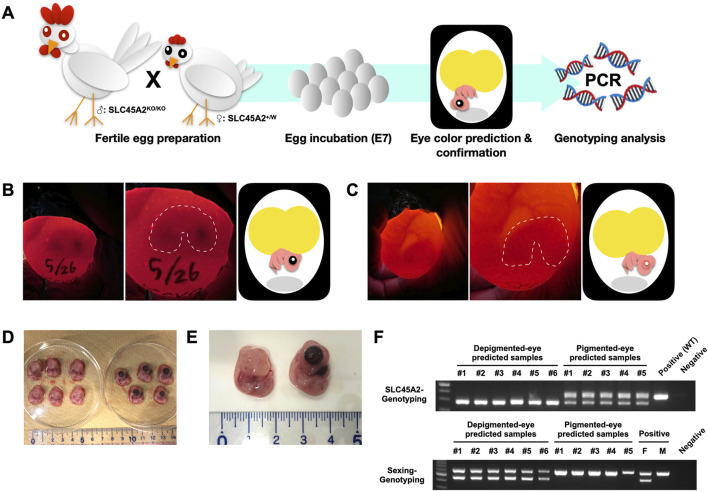
A practical application of simple in-ovo sexing in SLC45A2 knockout-derived eggs. **(A)** Illustration of the in-ovo sexing process, including the preparation of fertile eggs from SLC45A2 knockout chickens, incubation, embryonic eye color prediction and confirmation, and PCR-based genotyping analysis. **(B)** An egg predicted to carry an embryo with pigmented eyes during egg candling. The dotted line marks the shape and location of the embryo, with a black spot indicating a pigmented eye. The cartoon on the right depicts the position and status of the embryo within the whole egg. **(C)** An egg predicted to carry an embryo with depigmented eyes during egg candling. The dotted line marks the shape and location of the embryo, with no black spot visible in the region. The cartoon on the right illustrates the position and status of the embryo within the whole egg. **(D)** Embryos taken out from depigmented-eye-predicted eggs (left) and pigmented-eye-predicted eggs (right). **(E)** Distinct pigmentation differences between embryos from depigmented-eye-predicted eggs (left) and pigmented-eye-predicted eggs (right). **(F)** Gel electrophoresis results for SLC45A2 genotyping and Sex-Genotyping of the predicted samples. Six samples from depigmented-eye-predicted embryos and five from pigmented-eye-predicted embryos were subjected to both PCR tests. In SLC45A2 genotyping, all depigmented-eye samples displayed only the 281-bp product, whereas all pigmented-eye samples presented both 352-bp and 281-bp products. A wild-type (WT) sample served as a positive control for the 352-bp product. In sexing PCR, the female control produced 600-bp and 450-bp products, whereas the male control produced only the 450-bp product when standard avian sexing primers (2550F and 2718R) were used (Fridolfsson and Ellegren, 1999). All the depigmented-eye-predicted samples presented a female pattern, and all the pigmented-eye-predicted samples presented a male pattern.

Finally, we evaluated whether the SLC45A2 knockout chickens can be used for in-ovo sexing based on eye color. To mimic practical applications in industry, we designed a cross-combination to ensure the resulting offspring would carry only a hemizygous knockout in females and a heterozygous knockout in males. This allows the eye pigmentation phenotype to reflect sex perfectly. Homozygous knockout roosters (SLC45A2^KO/KO^) were crossed with wild-type hens, and their fertile eggs were incubated until E7. Eye color was predicted from outside the shell via simple lighting and then confirmed by opening the shell. The SLC45A2 genotype and sex were investigated through PCR analysis (Figure 5A).

We examined 11 fertile eggs, predicting six embryos containing pigmented eyes and five embryos without pigmented eyes (Figures 5B,C; S1 and S2 Movie). The predictions were confirmed by observing the embryos (Figures 5D,E), which achieved 100.0% accuracy (Table 3).

**TABLE 3:** Confirmation results for the predicted samples.

Eye color predicted by egg candling		Confirmed eye color	SLC45A2 genotype	Sex
Pigmented-eye predicted	#1	Pigmented	Heterozygous knockout	Male
	#2	Pigmented	Heterozygous knockout	Male
	#3	Pigmented	Heterozygous knockout	Male
	#4	Pigmented	Heterozygous knockout	Male
	#5	Pigmented	Heterozygous knockout	Male
	#6	Pigmented	Heterozygous knockout	Male
	Accuracy	100.0% (6/6)	100.0% (6/6)	100.0% (6/6)
Depigmented-eye predicted	#1	Depigmented	Hemizygous knockout	Female
	#2	Depigmented	Hemizygous knockout	Female
	#3	Depigmented	Hemizygous knockout	Female
	#4	Depigmented	Hemizygous knockout	Female
	#5	Depigmented	Hemizygous knockout	Female
	Accuracy	100.0% (5/5)	100.0% (5/5)	100.0% (5/5)

Next, we examined their genotype via SLC45A2-Genotyping (Figure 5F). Embryos without pigmented eyes produced a 281-bp PCR, confirming the 71-bp deletion, whereas embryos with pigmented eyes produced both 281-bp and 352-bp products, reflecting the knockout allele and the wild-type, respectively (Figure 5F). Moreover, Sexing-Genotyping PCR confirmed that all the depigmented-eye embryos were female and that all the pigmented-eye embryos were male, indicating 100.0% accuracy in genotype and sex prediction (Table 3). These findings demonstrate that in-ovo sexing with SLC45A2 knockout-derived eggs is a simple and highly accurate method for predicting chick embryonic sex.

## 4 Discussion

In this study, we generated SLC45A2 knockout chickens whose eyes did not show eye pigmentation in the embryonic stage. Male and female embryos derived from intercrossing between SLC45A2 homozygous knockout males (SLC45A2^KO/KO^) and wild-type females (SLC45A2^+/W^) developed black and colorless eyes, respectively, which can be identified by simple lighting during early developmental stages.

The target gene in this study, SLC45A2 (also known as membrane-associated transporter protein (MATP)), is located in melanosomes, the organelles responsible for melanin synthesis, storage, and transport (Seiji et al., 1963; Newton et al., 2001). In mammals, it regulates the luminal pH, enabling the incorporation of copper ions into tyrosinase for melanosome maturation (Bin et al., 2015; Le et al., 2020). Mutations in SLC45A2 have been linked to pigmentation variations in hair, skin, and eyes across multiple species (Newton et al., 2001; Fukamachi et al., 2001; Mariat et al., 2003). In birds, SLC45A2 mutations cause phenotypes such as *imperfect albinism*, *silver*, and *cinnamon* in chickens and Japanese quails (Gunnarsson et al., 2007). Our research confirmed that SLC45A2 loss-of-function causes eye depigmentation during embryonic stages. Although this change is still discernible at hatching, it diminishes in adulthood. Since SLC45A2 is Z-linked and female birds are heterogametic (ZW), this mutation can serve as a non-invasive, early-stage in-ovo sexing marker without affecting reproductive performance.

In this study, we established a new method for identifying the sex of chicks on Day 7 of incubation. To prevent any possibility of embryos developing consciousness and experiencing pain, the initial German legislation mandated that all fertilized eggs be sexed before Day 7 of incubation. However, further research commissioned by the German government concluded that chicken embryos do not feel pain before Day 13. Consequently, the law was amended to allow in-ovo sexing up to Day 12 (The Poultry Site, 2024). This revision was a positive development for the in-ovo sexing industry, as many technologies are effective before Day 13 but not before Day 7. Currently, major industry players offer detection from Day 9 onward. In our practical application, we used E7 (Day 7) eggs for sex detection and achieved perfect accuracy. Retinal pigmentation in chicken embryos begins around Hamburger‒Hamilton (HH) stage 22, approximately 3.5 days in incubation (Hamburger and Hamilton, 1992). We observed that eye color differences between pigmented and depigmented embryos could be distinguished as early as E5, indicating the potential for earlier sex detection. Earlier detection could improve flexibility in chick production workflows within the industry.

Unlike other genetically modified chick sexing techniques (Doran et al., 2018; Cinnamon and Cohen, 2020), our approach does not involve the insertion of exogenous genes but instead relies on targeted mutagenesis of the endogenous chicken SLC45A2 gene. Consequently, this strategy can be classified as a site-directed nuclease type-1 (SDN-1) modification, which may be exempt from GMO regulations in certain jurisdictions following appropriate regulatory assessment (e.g., Japan). Importantly, we employed simple egg candling for non-invasive detection, thereby minimizing contamination risk and eliminating the need for costly equipment typically associated with current in-ovo sexing technologies. Although several alternative methods have been reported, many rely on invasive sampling procedures or complex instrumentation (Xie et al., 2023; Corion et al., 2023). Because egg candling is already routinely implemented in large-scale hatchery operations, our method could be more readily integrated into existing industrial workflows, potentially reducing operational costs while improving accessibility.

Moreover, this targeted mutagenesis approach can be applied to most chicken breeds and potentially other poultry species, given the conservation of the SLC45A2 gene on the Z chromosome across avian species. Currently, PGC-mediated strategies, which are more efficient than other gene-editing methods, have become the primary approach for genetic modification in chickens and quails (van de Lavoir et al., 2006; Park et al., 2020; Lee et al., 2019). With the successful establishment of avian PGC cultures across various species, genetic engineering is now more accessible (Whyte et al., 2015; Chen et al., 2019; Jung et al., 2021; Gessara et al., 2021; Doddamani et al., 2025). This broad applicability significantly enhances its potential impact on poultry production.

In conclusion, our study established a framework for generating and evaluating SLC45A2 knockout chickens, demonstrating that this gene is essential for eye pigmentation while having no detectable adverse effects on reproductive performance. The introduction of the SLC45A2 knockout resulted in depigmented eyes in hemizygous knockout females, enabling highly accurate in-ovo sexing through routine egg candling. PCR-based genotyping and sexing further validated these predictions, underscoring the reliability of the approach. However, this study represents a laboratory-scale proof-of-concept with a limited sample size for practical in-ovo sexing applications. Future work should focus on large-scale validation under commercial production conditions, as well as the development of automated, high-throughput detection systems compatible with industrial hatchery workflows, rather than manual visual inspection. Despite these limitations, the proposed strategy provides a non-invasive and efficient method for early embryonic sex prediction, with substantial potential for application in the poultry industry.

## Data Availability

The original contributions presented in the study are included in the article/Supplementary Material, further inquiries can be directed to the corresponding author.
