# Supplementary material for: Eye pigmentation–based in-ovo chicken sexing via precision breeding
Source: Front Bioeng Biotechnol. 2026 Apr 1;14:1785893. doi: 10.3389/fbioe.2026.1785893 (PMC13079324; doi:10.3389/fbioe.2026.1785893)
Supplement: Supplementary file 2 [file Table1.docx]

Supplementary Material

# Supplementary Tables

**Table S1: SLC45A2 knockout chickens and their performance in eye pigmentation and reproduction**

| Generation | Genotype | Sex | Total number | Eye pigmentation  (Number (%)) | Reproduction capable*  (Number (%)) |
| --- | --- | --- | --- | --- | --- |
| F1 | SLC45A2^KO/+^ | Male | 4 | 4 (100.0%) | 4 (100.0%) |
| F2 | SLC45A2^KO/+^ | Male | 2 | 2 (100.0%) | 2 (100.0%) |
|  | SLC45A2^KO/KO^ | Male | 2 | 0 (0.0%) | 2 (100.0%) |
|  | SLC45A2^KO/W^ | Female | 2 | 0 (0.0%) | 2 (100.0%) |
| F3 | SLC45A2^KO/+^ | Male | 2 | 2 (100%) | 2 (100.0%) |
|  | SLC45A2^KO/KO^ | Male | 2 | 0 (0.0%) | 2 (100.0%) |
|  | SLC45A2^KO/W^ | Female | 2 | 0 (0.0%) | 2 (100.0%) |

* The chicken was able to produce functional sperm in males and lay fertilized eggs normally in females.

**Table S2: crRNA oligos and sequences for nMAD7 expression plasmid construction**

| Oligo name | Sequence (5’-3’) |
| --- | --- |
| crRNA-1-forward | AGATTCACGCCAGTGCTGCTCAGTG |
| crRNA-1-reverse | AAAACACTGAGCAGCACTGGCGTGA |
| crRNA-2-forward | AGATACGTCTGCTCTCATGAAGATA |
| crRNA-2-reverse | AAAATATCTTCATGAGAGCAGACGT |
| crRNA-3-forward | AGATTCTTCATGAGAGCAGACGTCA |
| crRNA-3-reverse | AAAATGACGTCTGCTCTCATGAAGA |

**Table S3: Primer sets and sequences for PCR**

| Primer name | Sequence (5’-3’) | Product size (bp) | Purpose |
| --- | --- | --- | --- |
| SLC45A2_NGS_F1 | TCGTCGGCAGCGTCAGATGTGTATAAGAGACAGCAAGGAAGACATGGACAGCACAG | 345 | Amplicon sequence analysis (exon 1) |
| SLC45A2_NGS_R1 | GTCTCGTGGGCTCGGAGATGTGTATAAGAGACAGGATCACTGGCAGAACCTACCAC | 345 | Amplicon sequence analysis (exon 1) |
| SLC45A2_NGS_F2 | TCGTCGGCAGCGTCAGATGTGTATAAGAGACAGGTCACAATGAGAGTCTTTTTGGTTT | 352 (amplicon sequence analysis); 352 (SLC45A2-KO genotyping-WT); 281 (SLC45A2-KO genotyping-KO) | Amplicon sequence analysis (exon 2); SLC45A2-KO genotyping |
| SLC45A2_NGS_R2 | GTCTCGTGGGCTCGGAGATGTGTATAAGAGACAGAGTACACGGTGATTACAAAATCCAT | 352 (amplicon sequence analysis); 352 (SLC45A2-KO genotyping-WT); 281 (SLC45A2-KO genotyping-KO) | Amplicon sequence analysis (exon 2); SLC45A2-KO genotyping |
| 2550F | GTTACTGATTCGTCTACGAGA | 600 (male); 600+450 (female) | Sexing-PCR |
| 2718R | ATTGAAATGATCCAGTGCTTG | 600 (male); 600+450 (female) | Sexing-PCR |

**Table S4: Egg-laying number and laying rate in wild-type and knockout hens**

| Week-old | | 28^th^ week (7 days) | | 29^th^ week (7 days) | | 30^th^ week (7 days) | | 31^th^ week (7 days) | | 32^th^ week (7 days) | | 33^th^ week (7 days) | | 28^th^–33^th^ week (42 days) | |
| --- | --- | --- | --- | --- | --- | --- | --- | --- | --- | --- | --- | --- | --- | --- | --- |
| Group | #NO | Egg number | Laying rate (%) | Egg number | Laying rate (%) | Egg number | Laying rate (%) | Egg number | Laying rate (%) | Egg number | Laying rate (%) | Egg number | Laying rate (%) | Egg number | Laying rate (%) |
| Wild-type | 506 | 6 | 85.7 | 6 | 85.7 | 7 | 100.0 | 7 | 100.0 | 6 | 85.7 | 7 | 100.0 | 39 | 92.9 |
|  | 509 | 5 | 71.4 | 6 | 85.7 | 6 | 85.7 | 6 | 85.7 | 6 | 85.7 | 6 | 85.7 | 35 | 83.3 |
|  | 373 | 7 | 100.0 | 7 | 100.0 | 6 | 85.7 | 7 | 100.0 | 7 | 100.0 | 6 | 85.7 | 40 | 95.2 |
| Knockout | 505 | 7 | 100.0 | 7 | 100.0 | 7 | 100.0 | 6 | 85.7 | 8 | 114.3 | 7 | 100.0 | 42 | 100.0 |
|  | 507 | 7 | 100.0 | 7 | 100.0 | 6 | 85.7 | 7 | 100.0 | 6 | 85.7 | 6 | 85.7 | 39 | 92.9 |
|  | 512 | 7 | 100.0 | 7 | 100.0 | 6 | 85.7 | 7 | 100.0 | 7 | 100.0 | 6 | 85.7 | 40 | 95.2 |

# Supplementary Data

S1 Movie (separate file). Visualization of embryo shape and pigmented eye in the egg during routine egg candling.

S2 Movie (separate file). Visualization of embryo shape and depigmented eye in the egg during routine egg candling.
